# Reference Gene Selection for Quantitative Real-Time Reverse-Transcriptase PCR in Annual Ryegrass (*Lolium multiflorum*) Subjected to Various Abiotic Stresses

**DOI:** 10.3390/molecules23010172

**Published:** 2018-01-16

**Authors:** Qiuxu Liu, Xiao Qi, Haidong Yan, Linkai Huang, Gang Nie, Xinquan Zhang

**Affiliations:** 1 Department of Grassland Science, Animal Science and Technology College, Sichuan Agricultural University, Chengdu 611130, China; sicauliuqiuxu@163.com (Q.L.); huanglinkai@sicau.edu.cn (L.H.); nieganggrass@hotmail.com (G.N.); 2 National Animal Husbandry Service, Ministry of Agriculture, Beijing 100000, China; tq07mms@sina.com; 3 Department of Horticulture, Virginia Polytechnic Institute and State University, 490 West Campus Dr., Blacksburg, VA 24061, USA; yanhd@vt.edu

**Keywords:** reference gene, annual ryegrass, abiotic stress, qRT-PCR, *Lolium*

## Abstract

To select the most stable reference genes in annual ryegrass (*Lolium multiflorum*), we studied annual ryegrass leaf tissues exposed to various abiotic stresses by qRT-PCR and selected 11 candidate reference genes, i.e., 18S rRNA, E2, GAPDH, eIF4A, HIS3, SAMDC, TBP-1, Unigene71, Unigene77, Unigene755, and Unigene14912. We then used GeNorm, NormFinder, and BestKeeper to analyze the expression stability of these 11 genes, and used RefFinder to comprehensively rank genes according to stability. Under different stress conditions, the most suitable reference genes for studies of leaf tissues of annual ryegrass were different. The expression of the eIF4A gene was the most stable under drought stress. Under saline-alkali stress, Unigene14912 has the highest expression stability. Under acidic aluminum stress, SAMDC expression stability was highest. Under heavy metal stress, Unigene71 expression had the highest stability. According to the software analyses, Unigene14912, HIS3, and eIF4A were the most suitable for analyses of abiotic stress in tissues of annual ryegrass. GAPDH was the least suitable reference gene. In conclusion, selecting appropriate reference genes under abiotic stress not only improves the accuracy of annual ryegrass gene expression analyses, but also provides a theoretical reference for the development of reference genes in plants of the genus *Lolium*.

## 1. Introduction

Annual ryegrass (*Lolium multiflorum*) is cultivated in temperate and subtropical regions worldwide and is used in forage and livestock systems as silage and green fodder owing to its high palatability and digestibility [1,2]. It is one of the most widely cultivated plants in the world, and grows in warm and humid climates. It is mainly used for artificial grass, clipping, and grazing [3]. However, environmental degradation from natural challenges, saline-alkali, acidic aluminum, drought, and heavy metals threatens the production of annual ryegrass. To enable more detailed and in-depth studies of gene expression, it is necessary to identify appropriate reference genes.

Quantitative reverse transcription PCR (qRT-PCR) is widely used to determine the expression of genes in different plant tissues owing to its high sensitivity, specificity, accuracy, and repeatability [4]; it is a powerful technical method to detect gene expression levels. However, the accuracy of qRT-PCR is affected by the quality of RNA, the efficiency of RNA reverse transcription, the design of primers, the initial sample capacity, etc. [5]. To accurately detect differences in gene expression, a reference gene is used as the calibration standard [6]. The ideal reference gene should exhibit steady expression in all cells, physiological states, and sample types. However, no reference gene is expressed stably under various experimental conditions [7] and it is therefore important to choose stable reference genes for specific experimental conditions.

Recent studies have explored appropriate reference genes for different plant species, plant organs, and experimental conditions [8]. GAPDH is the most stable among different tissues in the precocious grass [9], but in *Brachypodium distachyon*, it is not suitable [10]. EF-1 alpha exhibits the most stable expression under abiotic stress, but in the tomato, it is the least stable under light stress [11]. During leaf development, 18S rRNA is the most reliable reference gene, while 18S rRNA is the least stable gene during floral development [12]. In black medic root tissue, GAPDH is the most stable gene [13], but in switchgrass root tissue, it is ranked last [14]. In the non-heading Chinese cabbage, for eight different tissues, including the root and stem at the third leaf stage, leaves after bolting, flower buds, petioles, stamens, pistils, and seed pods, PP2A had the greatest stability among thirteen candidate reference genes [15]. Under different experimental conditions, it is easy to obtain different results. For example, in potato, 18S rRNA is the most stable reference gene for biotic stress (late blight exposure), and L2 + Cyclophilin are the most stable genes for abiotic stress (salt and cold stress) [16]. Therefore, it is necessary to screen stable reference genes for different plant species and experimental treatments.

In order to accurately analyze the stability of candidate reference genes, we used four software packages for analysis. Each software package implements a unique statistical algorithm. GeNorm (ver.3.5) [17], a Visual Basic Application (VBA) tool for Microsoft Excel, was employed as a means of analyzing the expression stability of the candidate reference genes based on the principle that the expression ratio of two perfect reference genes should be constant under different treatments. The expression stability value M1 based on the average pairwise expression ratio was calculated by this method. NormFinder (ver.0.953) [18] is a VBA applet based on a variance estimation approach; it ranks candidate reference genes according to their stability under differential experimental conditions, and stable gene expression is indicated by low average expression stability values (M2) (Ferreira et al., 2013) [19]. BestKeeper (ver.1.0) [5] is an Excel-based software tool that analyzes the stability of candidate reference genes based on the coefficient of variation (CV) and the standard deviation (SD). Finally, RefFinder [20], a user-friendly comprehensive web-based tool, was developed for evaluating reference gene stability from extensive experimental datasets. Therefore, these four software packages were used for comprehensive analyses in this study.

Advances in molecular biology are expected to lead to the identification of more stable and novel genes as reference genes, rather than traditional screening approaches for reference gene identification. In this study, seven traditional reference genes and four Unigenes from transcriptome sequencing data in annual ryegrass were evaluated under different abiotic stress treatments by qRT-PCR. This study aims to examine annual ryegrass leaf tissue under abiotic stress to determine the most stable reference genes and provide a theoretical basis and data correction method for studies of gene expression under abiotic stress. These findings can also provide support for studies of the annual ryegrass genome in the future.

## 2. Results

### 2.1. Verification of PCR Amplicons, Primer Specificity, and Gene-Specific PCR Amplification Efficiency

**Figure 1:**
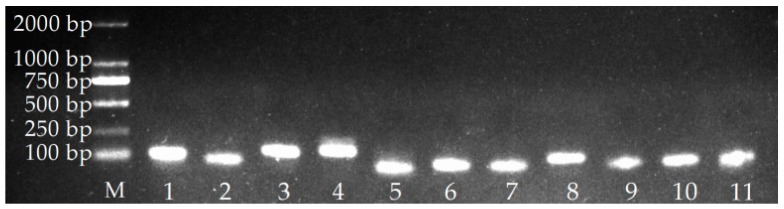
PCR products for 11 reference genes. M: DNA marker, 1: 18S rRNA, 2: E2, 3: eIF4A, 4: GAPDH, 5: HIS3, 6: SAMDC, 7: TBP-1, 8: Unigene71, 9: Unigene77, 10: Unigene755, 11: Unigene14912.

Using 1.5% agarose gel electrophoresis, fragments of 80–200 bp of HIS3, 18S rRNA, SAMDC, GAPDH, TBP-1, eIF4A, E2, Unigene71, Unigene77, Unigene755, and Unigene14912 clearly amplified, consistent with the expected fragment sizes, and no impurities or primer dimers were observed (Figure 1), indicating that the primers for the 11 candidate reference gene were highly specific and can be used for qRT-PCR analyses.

**Figure 2:**
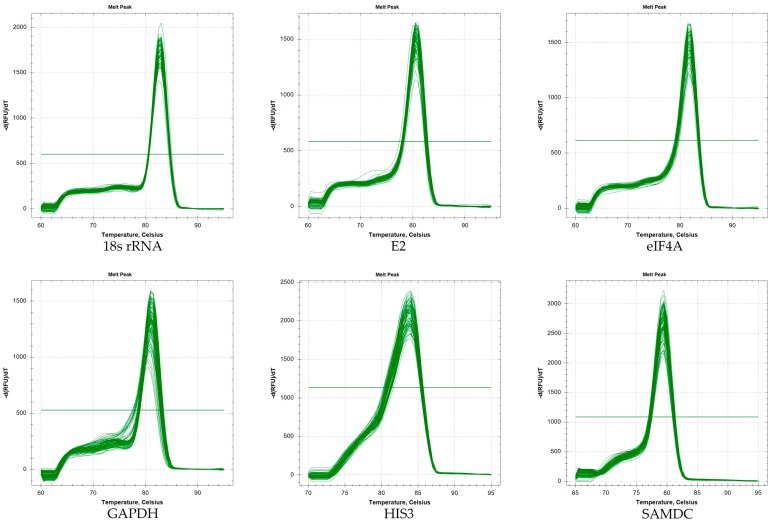

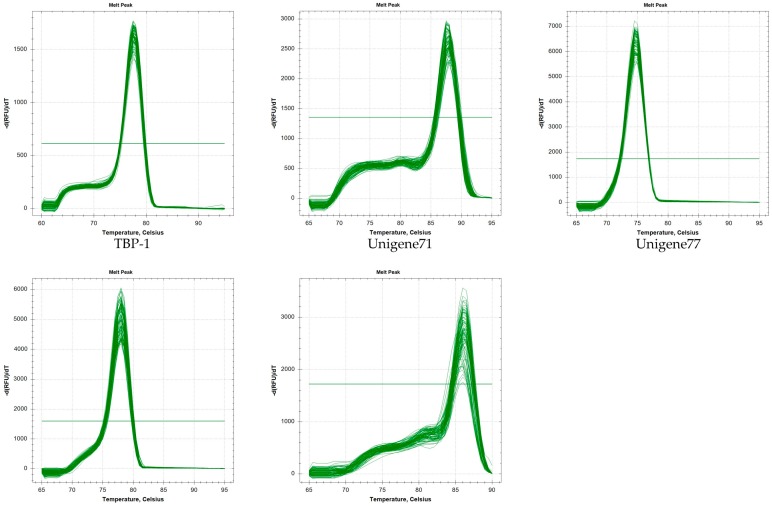
Melting curves for eleven genes.

Most of the candidate reference genes in various abiotic stress conditions exhibited melting curves with a single peak, showing that the primers are highly specific. Gene amplification curves for each sample had good repeatability, showing that the qRT-PCR results were accurate and reliable (Figure 2). However, in some samples, a miscellaneous peak of GAPDH indicated that the primer was not highly stable.

### 2.2. Analysis of the Expression Stability of Reference Genes under Different Types of Abiotic Stress

**Figure 3:**
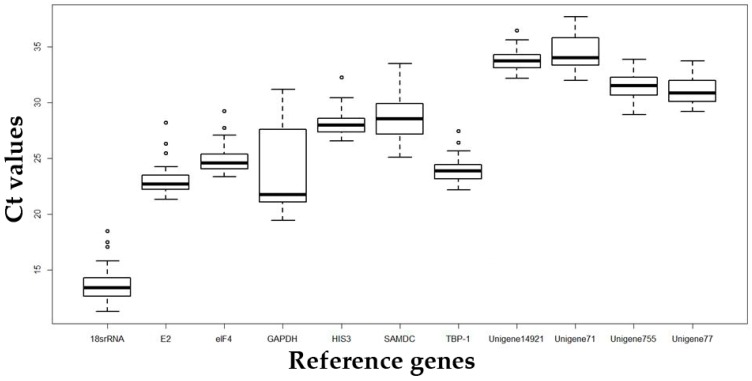
qRT-PCR *C*t values for all candidate reference genes in annual ryegrass leaf samples under various abiotic stress conditions. Variation is displayed as medians (lines), 25th to 75th percentiles (boxes), and ranges (whiskers).

Analysis of the raw expression levels across all samples identified variation among reference genes is shown in Figure 3. We present detailed data in Supplementary Table S1. *C*t values for the 11 genes ranged from 11.24 to 37.73, and the majority of these values were between 23.44 and 31.94. 18S rRNA was highly expressed compared to the other genes, reaching threshold fluorescence after only 13.6 amplification cycles, whereas the *C*t average of all reference genes within the datasets was approximately 27 cycles. As a result, the 18S rRNA transcripts were around 10,000-fold more abundant than the average for the dataset. Unigene71 and Unigene14912 transcripts were least abundant, with *C*t values of 34.03 and 33.75, respectively. The individual reference genes had different expression ranges across all studied RNA samples. However, before we performed the software analysis, we observed that GAPDH has the most discrete data and therefore its ranking should be lowest in subsequent analyses. By contrast, E2, eIF4A, HIS3, TBP-1, and Unigene14912 were expected to perform well in subsequent analyses. The wide expression ranges of the eleven tested reference genes confirmed that it is of the utmost importance to select a reliable reference gene to normalize gene expression under certain conditions.

### 2.3. Stability Ranking of Candidate Reference Genes for Different Abiotic Stresses

In this study, GeNorm, BestKeeper, NormFinder, and RefFinder were used to analyze the expression of relatively stable reference genes in different abiotic stress conditions. The results showed that reference gene exhibited expression differences depending on the abiotic stress.

#### 2.3.1. GeNorm Analysis

**Figure 4:**
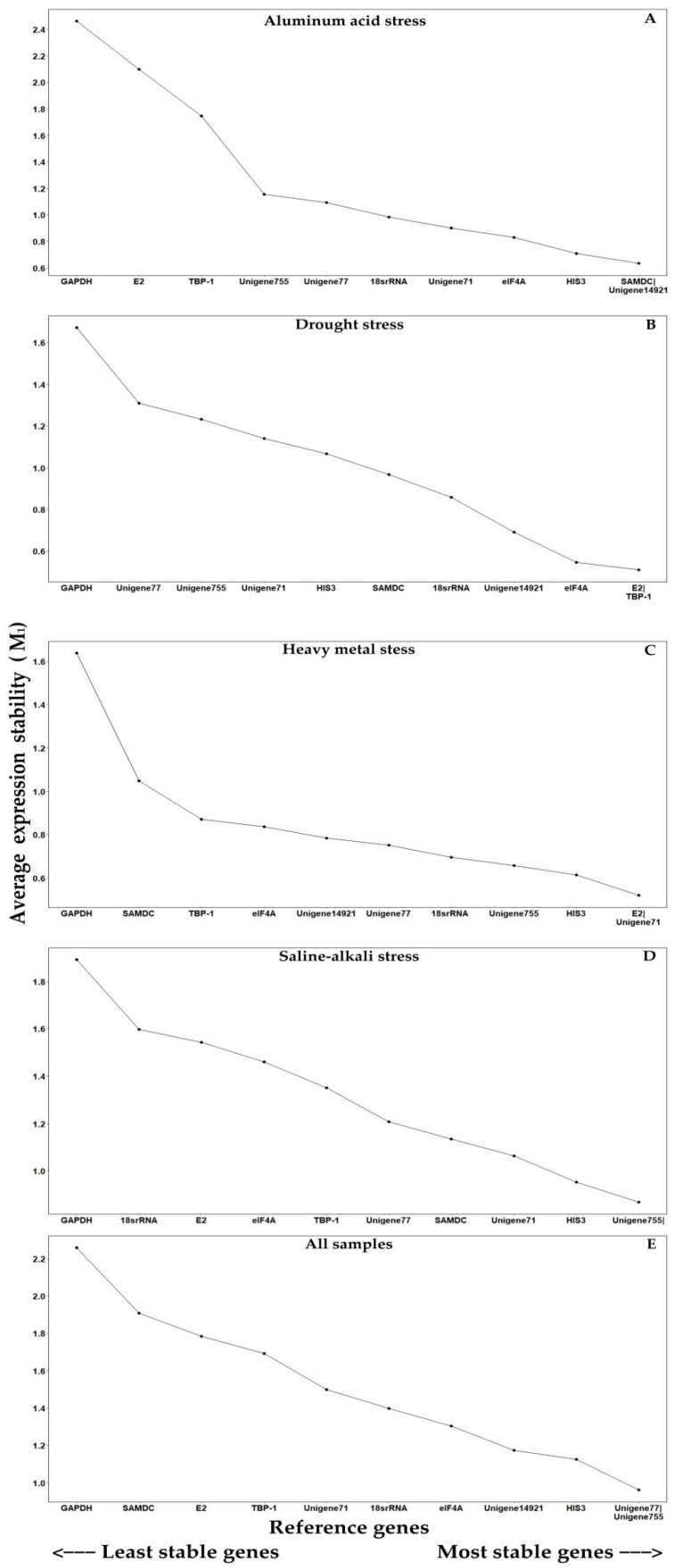
Average expression stability values (M_1_) of candidate reference genes by GeNorm analysis: (**A**) acidic aluminum stress exposure; (**B**) drought stress exposure; (**C**) heavy metal stress exposure; (**D**) saline-alkali stress exposure; (**E**) all samples.

GeNorm was used to rank the reference genes with respect to expression stability by calculating average pairwise expression ratios (M_1_), where values below 1.5 indicate stable expression. In this study, Unigene77 and Unigene755, with the same M_1_ value of 0.962, were the most stably expressed in all samples, while GAPDH, with an M_1_ value of 2.257, was the least stably expressed gene (Figure 4E). For the samples subjected to acidic aluminum stress, drought stress, heavy metal stress, and saline-alkali stress, the pairs SAMDC and Unigene14912, E2 and TBP-1, E2 and Unigene71, and Unigene755 and Unigene14912 ranked the highest, respectively, suggesting that these gene pairs were stable and could be used as references (Figure 4A–D).

**Figure 5:**
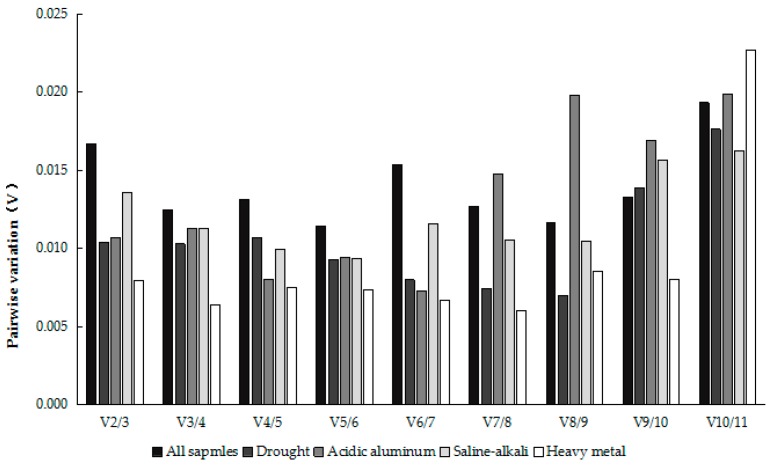
Pairwise variation (V) measure of the candidate reference genes. When V*_n_*/V*_n_*_+1_ < 0.15, then the optimal number of reference genes is N.

In some gene expression analysis of study, sometimes to express quantitatively the high precision requirements, or in some less obvious purpose gene expression quantity change, using a single reference genes cannot accord with the requirement of the experiment. Therefore, it has become a new criterion in quantitative PCR research to correct the expression of the target gene with two or more stable reference genes [21]. Accordingly, this study analyzed the pairwise variation of each reference gene (Figure 5), and the results showed that the pairwise variation of the two to eleven reference gene was less than 0.15. Vandesompele et al. [17] suggested that when the paired variation V value was less than 0.15, there was no need to add multiple reference genes for the correction. Therefore, we can see that the optimal number of reference genes is two.

#### 2.3.2. BestKeeper Analysis

BestKeeper was used to analyze the expression stability of genes by determining which genes had the lowest CV ± SD (Table 1). Our results indicated that Unigene71 had the lowest CV values under acidic aluminum and heavy metal stress; Unigene14912 showed the lowest CV values for drought stress, saline-alkali stress, and all samples. However, GAPDH showed the highest CV values of 14.04 ± 3.44, 11.84 ± 2.90, 14.26 ± 3.31 and 12.12 ± 2.82 when plant materials were subjected to acidic aluminum, drought, heavy metal, and saline-alkali stress, respectively, and had the highest CV values for all samples (Table 1).

#### 2.3.3. NormFinder Analysis

NormFinder was used to rank gene expression stability based on comparisons of the average pairwise variation of a gene to all other genes (M_2_). The stability values of reference genes were calculated by NormFinder and are shown in Table 2. Under different conditions, the lowest M_2_ values were assigned to different candidate reference genes. Unigene14912 ranked highest for all samples with an M_2_ value of 0.889. For acidic aluminum stress, 18S rRNA was the most-stable gene with an M_2_ value of 0.545. Under drought, heavy metal, and saline-alkali stress, eIF4A, Unigene71, and TBP-1 were the most suitable genes, with M_2_ values of 0.273, 0.26 and 0.455, respectively.

#### 2.3.4. RefFinder Analysis

Finally, RefFinder was used produce a comprehensive ranking of the most stable candidate reference genes under each stress condition (Table 3).

The expression levels of eIF4A, E2, and TBP-1 were most stable under drought stress (Table 3). Under saline-alkali treatment, Unigene14912, TBP-1, and HIS3 were most stable (Table 3). Under the stress of acidic aluminum, SAMDC, HIS3, and Unigene14912 were the most stable among the internal reference genes (Table 3). Under heavy metal stress, Unigene71, HIS3, and E2 were most stable (Table 3). 

### 2.4. Validation of the Usefulness of the Reference Genes Identified from This Study

To verify that reference genes influence qRT-PCR results for annual ryegrass leaves, the expression patterns of delta-1-pyrroline-5-carboxylate (P5CS1) and Cyt-Cu/Zn superoxide dismutase (Cyt-Cu/Zn SOD) were analyzed in varied abiotic stresses by using five different reference genes identified in previous results including three most stable reference genes, the combination of reference gene and the worst reference gene. P5CS1 is a critical gene for proline biosynthesis rate-limiting enzymes regulating the balance of osmotic and preventing the cell turgor pressure increases to adapt severe environment [22,23,24]. SOD is a kind of metalloenzyme commonly found in plants. When plants are under heavy metal stress, SOD is the first line of defense against oxidative damage [25]. SOD will work for hydrogen peroxide and oxygen molecules by disproportionation on superoxide anion, which is efficient removal of oxygen free radicals in plants and protects cells from oxidative damage [26]. Advanced plants have Cu/Zn SOD, which exists in the cytoplasm and chloroplasts [27].

**Figure 6:**
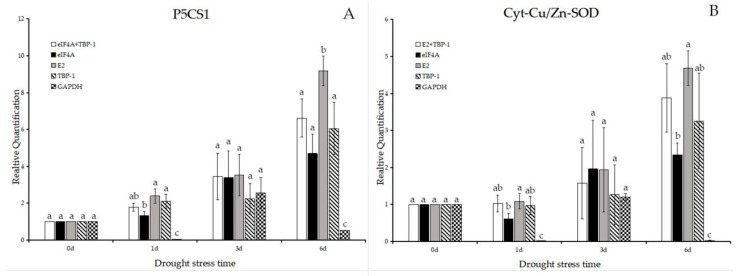

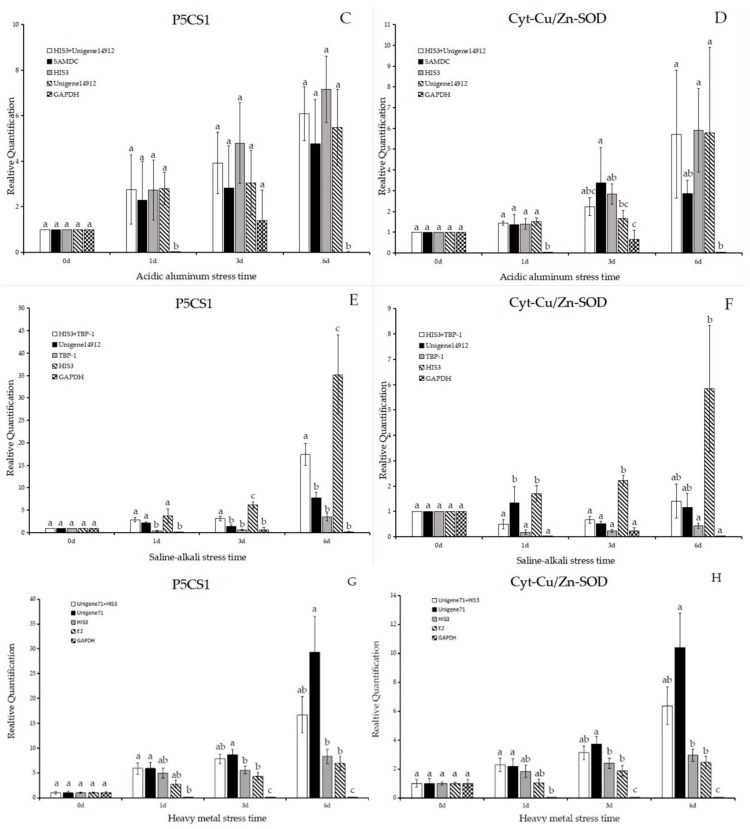
Expression levels of P5CS1 and Cyt-Cu/Zn SOD in annual ryegrass leaves under different stresses(drought stress, saline-alkali stress, acidic aluminum stress, heavy metal stress) at different times (days 0, 1, 3 and 6). (**A**–**H**) represent expression levels of P5CS1 and Cyt-Cu/Zn SOD. Bars indicate standard errors and different letters above the bars represent significant differences (*p* < 0.05).

We utilized the 2^−ΔΔ*C*t^ method to calculate the expression of P5CS1 and Cyt-Cu/Zn SOD under different stresses. As shown in Figure 6A,B, P5CS1 had same expression patterns using eIF4A + TBP-1, eIF4A, E2 and TBP-1 for normalization under drought stress. The similar results were obtained in Cyt-Cu/Zn SOD expression patterns. Specially, using TBP-1 for normalization, the expression of P5CS1 was induced with a 2-fold increase on the first day, 4-fold increase on the 3rd day, and 9-fold increase on the 6th day. 

Under acidic aluminum stress, P5CS1 and Cyt-Cu/Zn SOD had same expression patterns using HIS3 + Unigene14912, HIS3, and Unigene14912 for normalization (Figure 6C,D). Using Unigene14912 for normalization, the expression of P5CS1 was induced with a 3-fold increase on the first day, 5-fold increase on the 3rd day, and 7-fold increase on the 6th day, while the expression of Cyt-Cu/Zn SOD was induced with a 1.5-fold increase on the first day, 3-fold increase on the 3rd day, and 6-fold increase on the 6th day.

The HIS3 had the best efficient for analyzing the expression patterns of P5CS1 and Cyt-Cu/Zn SOD under saline-alkali stress. The expression of P5CS1 was induced with a 3-fold increase on the first day, a 5-fold increase on the 3rd day, and a 35-fold increase on the 6th day (Figure 6E,F). The similar results were obtained for Cyt-Cu/Zn SOD.

As the results show (Figure 6G,H) that P5CS1 had same expression patterns using Unigene71, HIS3, and E2 for normalization under heavy metal stress. The same results were obtained in Cyt-Cu/Zn SOD that both two target genes were up-regulated. Of note, using Unigene71 for normalization, the expression of P5CS1 was induced with a 5-fold increase on the first day, 8-fold increase on the 3rd day, and 29-fold increase on the 6th day.

## 3. Discussion

qRT-PCR has become an important technique for the analysis of gene expression owing to its sensitivity, accuracy, and high-throughput nature [28]. The accuracy of qRT-PCR depends on the stability of reference genes used for data normalization [29]. Under different conditions, changes in reference gene expression may lead to incorrect analyses of real-time data. Hence, it is important to identify suitable reference genes for gene expression analyses by qRT-PCR for each condition, rather than relying on reference genes from published data for different plant species [8].

With rapid developments in biotechnology, traditional reference genes have not met the needs of researchers. Accordingly, more stable reference genes are needed for gene expression analyses to detect very small differences in gene expression. In this study, seven traditional reference genes and four transcriptome Unigenes were analyzed to assess traditional reference genes and explore unknown and stable genes. We compared three different statistical applets, GeNorm, BestKeeper, and NormFinder to evaluate the 11 reference genes in annual ryegrass. The three applets yielded different rankings of stable reference genes depending on treatment (Table 3). It is worth noting that Unigene14912 is the most stable reference gene for all samples and under saline-alkali stress. Additionally, Unigene71 was the most stable of the reference gene under heavy metal stress. By contrast, the traditional reference genes SAMDC and eIF4A only performed well under acidic aluminum and drought stress, respectively. In this study, two new stable reference genes, Unigene14912 and Unigene71, were successfully identified, providing more potential reference genes for future studies.

BestKeeper and NormFinder identified Unigene14912 as the most stable reference gene, followed by eIF4A for all samples. GeNorm identified Unigene77 and Unigene755 as the best reference genes, followed by HIS3. GAPDH ranked last using all three methods. The most stable reference genes varied among the three methods used in this study, but the identification of unstable genes was consistent, and the inconsistent results may be explained by the different models associated with each statistical algorithm [30,31].

The results of this study were consistent with results obtained for other species. Three methods showed that under abiotic stress, annual ryegrass eIF4A was highly stable, similar to the results for closely related species, e.g., *Lolium perenne* under abiotic stress [32], darnel (*Lolium temulentum*) [33], and the Kentucky bluegrass [9], indicating that it is the most suitable for reference gene. GAPDH exhibits similar results across species, such as papaya (*Carica papaya*) [34], rice (*Oryza sativa*) [35], and tobacco (*Nicotiana tabacum*) [36], in which it was not suitable for use as a reference gene, but is involved in other biochemical reactions during abiotic stress.

The transcript abundances of Unigenes were generally low. In order to improve reliability of the result, the combination of traditional and novel reference genes are recommended. In the validation experiment under saline-alkali and heavy metal stress, the standard deviation of the combination of the reference gene for normalization is smaller than single reference gene, which indicating that the combination of the reference gene was more accurate and powerful in gene expression analysis. The most suitable reference gene combination was HIS3 + TBP-1 and Unigene71 + HIS3 under saline-alkali stress and heavy metal stress, respectively. 

The material we use is a single variety of leaves. Although it has been able to meet the research needs, it is not perfect. In future studies, we will explore between different varieties, different sites, and different developmental stages, reference genes perform excellent. 

## 4. Materials and Methods

### 4.1. Materials

This experimental subject is “Chuannong NO.1”, developed by the Sichuan Agricultural University (Chengdu, China). Seeds were sown in 25 × 19 × 6 cm pots and the matrix was silica sand. They were placed in an incubator with a light intensity of 100 μmol/(m·s), 25 °C and day/night cycles of 16 and 8 h. When the leaf counts of the plants reached 5–6, stress treatments were initiated. Under drought stress, plants were treated with 15% PEG6000 for 6 days. For acidic aluminum treatment, plants were treated with 0.1 mol/L AlCl_3_ (pH 2.9) for 6 days. For heavy metal treatment, the nutrient solution was filled with 200 mg/L Cr^6+^ Hoagland’s for 6 days. For saline-alkali stress, plants were treated with 0.1 mol/L NaHCO_3_ (pH 8.2) for 6 days. We take a sample of several seedlings (upper leaves) in the same pot. Take three mixed samples at four time points (0, 1, 3, 6 days) under four treatments (drought, saline-alkali, acidic aluminum, heavy metal). There are three biological repeats for each sampling point. We treat the before treatment (0 day) as a control group in each treatment, as described by Huang L et al. [37]. All samples frozen in liquid nitrogen, and stored at −80 °C for later use. The 48 annual ryegrass samples during the study and which datasets they were included in Supplementary Table S2.

### 4.2. Reagents and Instruments

We used the following reagents. RNA extraction reagent from the RNA Simple Total RNA Kit (TianGen Biotech Co., Ltd., Beijing, China), reverse transcriptase reagent from the iScript cDNA Synthesis Kit (Bio-Rad Laboratories Inc., Hercules, CA, USA), and quantitative fluorescence PCR kitswere purchased from TAKARA (Shiga, Japan). Primers were obtained from Sangon Biological Engineering (Shanghai), Beijing branch (China). A fluorescence quantitative instrument was used for fluorescence quantitative PCR (CFX-96; Bio-Rad).

### 4.3. RNA Extraction and First-Strand cDNA Synthesis

The RNA simple Total RNA Kit (TianGen Biotech Co., Ltd., Beijing, China) was used to extract RNA from annual ryegrass material and RNase-free DNase I (GBC, Beijing, China) was used to remove DNA. The Ultramicro Spectrophotometer (NanoVue Plus Spectrophotometer, Wilmington, DE, USA) was used to detect RNA purity and concentration at A260 nm/A280 nm and A260 nm/A230 nm. After 1% agarose gel electrophoresis, the iScript cDNA Synthesis Kit (Bio-Rad Laboratories Inc.) was used to reverse transcribe total RNA samples into cDNA, and samples were diluted 1:5 with ultrapure water and stored at −80 °C for later use.

### 4.4. Selection of Reference Genes and PCR Primer Design

Six common reference genes from perennial ryegrass (*Lolium perenne* L.) (18S rRNA, SAMDC, GAPDH, E2, eIF4A, and TBP-1) and four Unigenes obtained from annual ryegrass transcriptome data [38] (Unigene71, Unigene77, Unigene755, and Unigene14912) were selected as candidate reference genes. For HIS3, available expressed sequence tags of *Betula luminifera* H. Winkl were used. The target genes P5CS1 and Cyt-Cu/Zn SOD were obtained from my group [39]. We used these gene sequences for cloning, followed by blast searches and primer design using the online tool Primer3 (http://frodo.wi.mit.edu/primer/) (Table 4). The primer design conditions were as follows: Tm, 58–62 °C; PCR product length, 75–150 bp; Length of primers, 18–24 bp; GC content, 45–55%. The amplification efficiency of all primers ranged from 87.1% (18S rRNA) to 108% (HIS3) (Table 4).

### 4.5. qRT-PCR Amplification Procedure

In a fluorescence quantitative PCR tube (TLS-0851; Bio-Rad), 2 μL of cDNA (30 ng/μL), 1.5 μL ofreverse primer (10 μmol/L), 1.5 μL of forward primer (10 μmol/L), 10 μL 2 × SYBR Premix Ex Taq (5 U/μL), and 5 μL of ddH_2_O were added for a total volume of 20 μL. The procedure was repeated 3 times for each biological sample and 4 times for each technology. The amplification procedure was as follows: 95 °C for 30 s, followed by 95 °C for 5 s, and 64 °C for 30 s, repeated 40 times. Then, an extension phase was performed from 60 °C to 95 °C, where the temperature for each cycle increased by 0.5 °C for 5 s to obtain Tm and fluorescent signals for the melting curve. All qRT-PCRs were run in quadruplicate for technical samples and triplicate for biological samples.

### 4.6. Data Analysis

qRT-PCR analyses and GeNorm, NormFinder, and BestKeeper were used to analyze the stability of 11 candidate reference genes. Finally, RefFinder was used to sequence the 11 reference genes and select the gene with the highest stability under different stress conditions. The top three ranked genes for heavy metal stress and lowest ranked gene were used to calculate P5CS1 and Cyt-Cu/Zn SOD gene expression to evaluate the effectiveness of the reference genes.

## 5. Conclusions

In this study, the expression levels of 11 candidate reference genes under various types of stress were analyzed in annual ryegrass. For different abiotic stresses, the most stable reference genes differed. The eIF4Aexpression was most stable under drought stress; the most appropriate reference gene is HIS3+TBP-1 under saline-alkali stress; the expression of HIS3 is best suited for the reference gene under acidic aluminum stress; Unigene71 + HIS3 is the optimal choice of reference gene under heavy metal stress. Unigene14912 was the best gene when considering all samples. The four treatments differed with respect to the most stable gene, suggesting that no single gene can be consistently used for data normalization across different conditions [40], but we found that HIS3, Unigene14912, E2 and TBP-1 were stable in two or more treatments. Based on the analysis of GeNorm software for pairwise variation, we concluded that the combination of Unigene14912 and HIS3 should be selected as the reference gene. The study results indicate genes that can be used as suitable candidate reference genes under different stresses in annual ryegrass, and provide a basis for Lolium expression analyses.

## Figures and Tables

**Table 1:** Expression stability values for annual ryegrass candidate reference genes calculated using BestKeeper.

Rank	Acidic Aluminum Stress	Drought Stress	Heavy Metal Stress	Saline-Alkali Stress	All Samples
1	Unigene71	Unigene14912	Unigene71	Unigene14912	Unigene14912
	(1.48 ± 0.49)	(1.75 ± 0.58)	(0.86 ± 0.29)	(2.43 ± 0.82)	(2.14 ± 0.72)
2	eIF4A	Unigene71	HIS3	HIS3	Unigene755
	(1.76 ± 0.43)	(1.98 ± 0.71)	(1.56 ± 0.43)	(2.46 ± 0.71)	(2.95 ± 0.93)
3	Unigene14912	eIF4A	Unigene755	Unigene71	HIS3
	(1.9 ± 0.64)	(2.29 ± 0.55)	(1.56 ± 0.49)	(2.59 ± 0.93)	(3.08 ± 0.87)
4	HIS3	E2	E2	SAMDC	eIF4A
	(2.01 ± 0.56)	(2.68 ± 0.61)	(2.09 ± 0.47)	(2.78 ± 0.78)	(3.35 ± 0.83)
5	SAMDC	TBP-1	Unigene14912	TBP-1	Unigene77
	(2.41 ± 0.71)	(3.02 ± 0.72)	(2.26 ± 0.77)	(3.45 ± 0.86)	(3.4 ± 1.06)
6	Unigene755	SAMDC	Unigene77	Unigene755	Unigene71
	(2.51 ± 0.79)	(3.27 ± 0.87)	(2.3 ± 0.71)	(3.59 ± 1.13)	(3.69 ± 1.27)
7	Unigene77	HIS3	TBP-1	Unigene77	TBP-1
	(2.63 ± 0.80)	(4.14 ± 1.19)	(2.44 ± 0.57)	(3.96 ± 1.25)	(4.39 ± 1.06)
8	18S rRNA	Unigene755	eIF4A	eIF4A	E2
	(6.46 ± 0.84)	(4.14 ± 1.30)	(2.62 ± 0.65)	(4.11 ± 1.06)	(5.2 ± 1.21)
9	TBP-1	Unigene77	SAMDC	E2	SAMDC
	(8.25 ± 2.03)	(4.23 ± 1.32)	(3.78 ± 1.15)	(5.15 ± 1.25)	(5.22 ± 1.50)
10	E2	18S rRNA	18S rRNA	18S rRNA	18S rRNA
	(8.83 ± 2.06)	(8.82 ± 1.17)	(5.09 ± 0.67)	(9.7 ± 1.46)	(8.57 ± 1.17)
11	GAPDH	GAPDH	GAPDH	GAPDH	GAPDH
	(14.04 ± 3.44)	(11.84 ± 2.90)	(14.26 ± 3.31)	(12.12 ± 2.82)	(13.36 ± 3.19)

Note: expression stability and ranking of 11 candidate reference genes calculated with BestKeeper under all sample conditions, acidic aluminum, drought, heavy metal, and saline-alkali stress. The stability of eleven reference genes was identified based on the coefficient of variation (CV) and standard deviation (SD).

**Table 2:** Expression stability values for annual ryegrass candidate reference genes calculated using NormFinder.

Rank	Acidic Aluminum Stress	Drought Stress	Heavy Metal Stress	Saline-Alkali Stress	All Samples
1	18S rRNA	eIF4A	Unigene71	TBP-1	Unigene14912
	(0.545)	(0.273)	(0.26)	(0.445)	(0.899)
2	SAMDC	E2	HIS3	Unigene14912	eIF4A
	(0.843)	(0.594)	(0.374)	(0.764)	(0.928)
3	Unigene71	Unigene14912	TBP-1	eIF4A	HIS3
	(0.955)	(0.606)	(0.426)	(0.892)	(1.003)
4	HIS3	TBP-1	eIF4A	Unigene755	Unigene755
	(0.962)	(0.734)	(0.483)	(1.043)	(1.074)
5	Unigene14912	HIS3	Unigene755	HIS3	18S rRNA
	(1.092)	(0.874)	(0.511)	(1.195)	(1.162)
6	eIF4A	SAMDC	E2	E2	Unigene77
	(1.207)	(0.891)	(0.550)	(1.232)	(1.318)
7	Unigene755	Unigene71	18S rRNA	18S rRNA	Unigene71
	(1.417)	(1.122)	(0.895)	(1.319)	(1.562)
8	Unigene77	Unigene755	Unigene77	Unigene77	TBP-1
	(1.425)	(1.129)	(0.976)	(1.474)	(1.684)
9	TBP-1	18S rRNA	Unigene14912	SAMDC	E2
	(3.224)	(1.327)	(1.015)	(1.487)	(1.803)
10	E2	Unigene77	SAMDC	Unigene71	SAMDC
	(3.319)	(1.472)	(1.604)	(1.693)	(2.062)
11	GAPDH	GAPDH	GAPDH	GAPDH	GAPDH
	(3.707)	(3.174)	(4.223)	(3.064)	(3.564)

Note: expression stability and ranking of 11 candidate reference genes were calculated with NormFinder under all samples, acidic aluminum, drought, heavy metal and saline-alkali stress. Lower average stability (M_2_ value) indicates more stable expression.

**Table 3:** Stability ranking of candidate reference genes.

Method	Stability (High→Low)										
	1	2	3	4	5	6	7	8	9	10	11
All samples											
BestKeeper	Unigene14912	eIF4A	HIS3	Unigene755	Unigene77	TBP-1	18S rRNA	E2	Unigene71	SAMDC	GAPDH
NormFinder	Unigene14912	eIF4A	HIS3	Unigene755	18S rRNA	Unigene77	Unigene71	TBP-1	E2	SAMDC	GAPDH
GeNorm	Unigene77|Unigene755		HIS3	Unigene14912	eIF4A	18S rRNA	Unigene71	TBP-1	E2	SAMDC	GAPDH
RefFinder	Unigene14912	HIS3	eIF4A	Unigene755	Unigene77	18S rRNA	TBP-1	Unigene71	E2	SAMDC	GAPDH
Drought stress											
BestKeeper	eIF4A	Unigene14912	E2	Unigene71	TBP-1	SAMDC	18S rRNA	HIS3	Unigene755	Unigene77	GAPDH
NormFinder	eIF4A	E2	Unigene14912	TBP-1	HIS3	SAMDC	Unigene71	Unigene755	18S rRNA	Unigene77	GAPDH
GeNorm	E2|TBP-1		eIF4A	Unigene14912	18S rRNA	SAMDC	HIS3	Unigene71	Unigene755	Unigene77	GAPDH
RefFinder	eIF4A	E2	TBP-1	Unigene14912	SAMDC	HIS3	Unigene71	18S rRNA	Unigene755	Unigene77	GAPDH
Saline-alkali stress											
BestKeeper	HIS3	SAMDC	Unigene14912	TBP-1	Unigene71	eIF4A	Unigene755	Unigene77	E2	18S rRNA	GAPDH
NormFinder	TBP-1	Unigene14912	eIF4A	Unigene755	HIS3	E2	18S rRNA	Unigene77	SAMDC	Unigene71	GAPDH
GeNorm	Unigene755|Unigene14912		HIS3	Unigene71	SAMDC	Unigene77	TBP-1	eIF4A	E2	18S rRNA	GAPDH
RefFinder	Unigene14912	TBP-1	HIS3	Unigene755	eIF4A	SAMDC	Unigene71	E2	Unigene77	18S rRNA	GAPDH
Acidic aluminum stress											
BestKeeper	eIF4A	Unigene71	HIS3	Unigene14912	SAMDC	Unigene755Unigene77		18S rRNA	TBP-1	E2	GAPDH
NormFinder	18S rRNA	SAMDC	Unigene71	HIS3	Unigene14912	eIF4A	Unigene755	Unigene77	TBP-1	E2	GAPDH
GeNorm	SAMDC|Unigene14912		HIS3	eIF4A	Unigene71	18S rRNA	Unigene77	Unigene755	TBP-1	E2	GAPDH
RefFinder	SAMDC	HIS3	Unigene14912	eIF4A	18S rRNA	Unigene71	Unigene755	Unigene77	TBP-1	E2	GAPDH
Heavy metal stress											
BestKeeper	Unigene71	HIS3	E2	Unigene755	TBP-1	eIF4A	18S rRNA	Unigene77	Unigene14912	SAMDC	GAPDH
NormFinder	Unigene71	HIS3	TBP-1	eIF4A	Unigene755	E2	18S rRNA	Unigene77	Unigene14912	SAMDC	GAPDH
GeNorm	E2|Unigene71		HIS3	Unigene755	18S rRNA	Unigene77	Unigene14912	eIF4A	TBP-1	SAMDC	GAPDH
RefFinder	Unigene71	HIS3	E2	Unigene755	TBP-1	eIF4A	18S rRNA	Unigene77	Unigene14912	SAMDC	GAPDH

**Table 4:** Primer sequences for eleven reference genes used in the real-time qRT-PCR analysis.

Genes Name	Accession ID	Genes Description	Primer Sequence (5′→3′) Forward and Reverse	Tm/°C	Amplicon Length/bp	Amplification Efficiency	*R*^2^
18S rRNA	NC003071.7	18S ribosomal RNA	F:GATAGGAAGAGCCGACAT R:ATACGAACCGTGAAAGCG	58.5	156	87.1%	0.999
SAMDC	HO177535	*S*-adenosylmethionine decarboxylase	F:TAGCCTGTTCATCTACTC R:AAGAATCCTTGGAATGGT	55	82	92.2%	0.982
GAPDH	GR518033	Glyceraldehyde-3-phosphate dehydrogenase	F:TCTGACCGTTAGACTTGAGAAGG R:CTTGAGCTTACCCTCAGACTCCT	59	135	100.8%	0.991
TBP-1	GO924783	Proteasome regulatory subunit 6A homolog	F:TGCTTAGTTCCCCTAAGATAGTGA R:CTGAGACCAAACACGATTTCA	60	112	104.2%	0.993
eIF4A	GO924770	Eukaryotic initiation factor4 alpha	F:AACTCAACTTGAAGTGTTGGAGTG R:AGATCTGGTCCTGGAAAGAATATG	59	168	95.4%	0.991
E2	GO924794	Ubiquitin-conjugatingenzyme	F:CGGTTCTGTGCCAAAATGT R:CAGCTATCTCCAACGGTTCA	61.6	111	92.9%	0.990
HIS3	KM454863.1	Imidazoleglycerol-phosphate dehydratase	F:ACTTCAAGACTGATCTGCGTTTCC R:CATGGATGGCACAAAGGTTGG	63.3	80	108%	0.994
Unigene71		unknown	F:CTTGGAGGAAGAGGGACGAG R:GAGACAGGAGAGAGTGGCGG	61.3	134	93.4%	0.992
Unigene77		unknown	F:CAGGTAAAAGGAACAAACAT R:TCAATTCAGTAATCAAAGGC	56.9	98	89.6%	0.980
Unigene755		unknown	F:AAATGATAAACTGACCAAAC R:CCACTCAAGAGCCAAAATAG	55	101	91.3%	0.980
Unigene14912		unknown	F:TACACCCATGAGAATGCCAC R:TGTTACCTTGAGGAGTCGGA	59.4	110	91.2%	0.982
P5CS1	JX470539	Delta-1-pyrroline-5-carboxylate	F:ATAACCAATGCTATCCCTGAC R:TCTTAGTCGTTGCCTTGA	51	160		
Cyt-Cu/Zn SOD	JQ269677	Cytosolic Cu/Zn superoxide	F:GGCTGAGTATCCCATTT R:CTGCCTTTGCTGTTCT	57	87		

## Supplementary Materials

The supplementary materials are available online, Table S1: The *C*t values was collected from each treatment and time point by triplicate for biological, Table S2: The 48 annual ryegrass samples during the study and which datasets they were included in.
